# First person – Joshua Ginzel

**DOI:** 10.1242/dmm.052337

**Published:** 2025-03-19

## Abstract

First Person is a series of interviews with the first authors of a selection of papers published in Disease Models & Mechanisms, helping researchers promote themselves alongside their papers. Joshua Ginzel is first author on ‘ Nonlinear progression during the occult transition establishes cancer lethality’, published in DMM. Joshua is a PhD student in the lab of Joshua Snyder at Duke University, Durham, NC, USA, investigating cancer initiation and progression using systems biology approaches in mouse models.


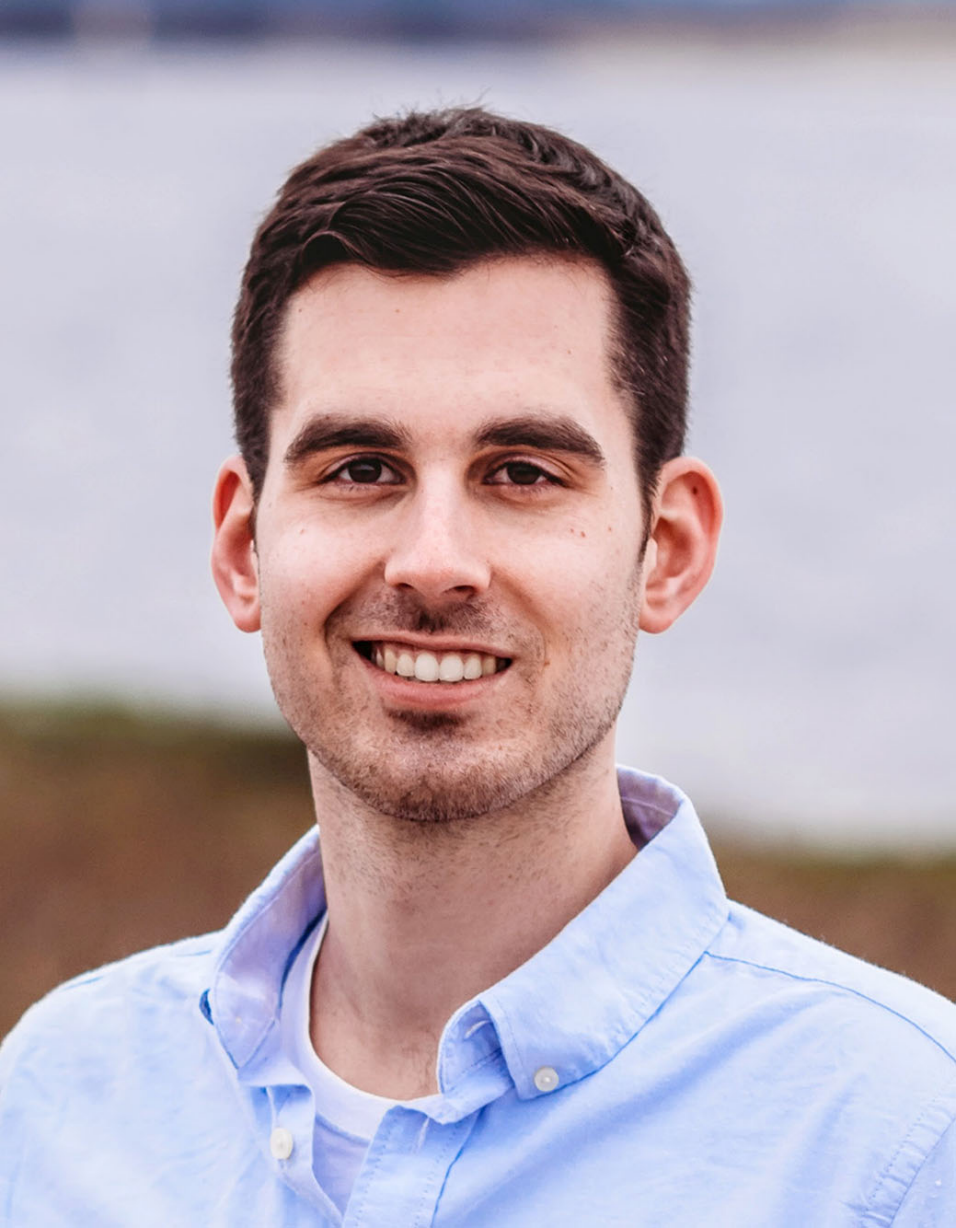


**Joshua Ginzel**

**Who or what inspired you to become a scientist?**

I have always had an affinity for fixing things and a natural curiosity for biology, which led me to believe that I wanted to become a doctor. While in my freshmen year of college, I was in a program that exposed me to research for the first time by allowing me to rotate through a few labs on campus. The passion of the professors and other undergraduate students for their chosen research fields opened my eyes to academic research, and I realized that I actually enjoyed learning why something was broken more than fixing it. Once I began my own research, I was captivated by understanding how development works and especially how it intersects with and impacts disease at a systems level.

**What is the main question or challenge in disease biology you are addressing in this paper? How did you go about investigating your question or challenge?**

All cancer screening is based on the paradigm that cancer growth and progression occurs in a linear fashion. The introduction of early screening in breast cancer has presented a significant paradox wherein the number of early-stage breast cancers has dramatically increased, yet very few metastatic cancers are prevented. This suggests that progression to lethal metastasis is not necessarily correlated with growth rate. One of the most significant obstacles to studying this process is the lack of tractable preclinical models in which a cancer can be observed throughout the various stages of the disease. When characterizing our cancer rainbow model of HER2 breast cancer, we observed differences in phenotypic changes occurring during mammary gland development and tumorigenic potential between the three isoforms of HER2 that seemed to recapitulate the heterogeneity of the human disease. However, in order to study tumor progression, we would need to collect a significant amount of longitudinal data. Leveraging high-throughput imaging techniques, we collected many discrete data points from our mouse model across time and used these data to inform a dynamic mathematical model of tumor progression in order to find critical transition rates and metastatic behaviors.

**How would you explain the main findings of your paper to non-scientific family and friends?**

Currently, breast cancers are detected as early as possible by mammography in order to treat before the cancer can become lethal. However, a significant number of patients will still progress to lethal disease after early treatment. Using a mouse model of breast cancer and mathematical modeling, we traced two types of breast cancer from one single cell to screen detectable tumors and beyond in order to correlate critical transition points of tumor growth with the ability to metastasize to distant organs. We show that some tumors grow to become screen detectable before spreading to distant organs, while other tumors are able to spread before the tumor is large enough to detect. These tumors that spread early represent the worst-case scenario in breast cancer screening where, despite early detection and treatment, progression to metastasis is inevitable.

Our model allows direct comparisons between cancers that can be detected and intercepted and those that become lethal long before current clinical intervention can occur.

**What are the potential implications of these results for disease biology and the possible impact on patients?**

We believe that these data demonstrate in a quantitative and experimental model why breast cancer screening is so challenging. Our model allows direct comparisons between cancers that can be detected and intercepted and those that become lethal long before current clinical intervention can occur. By showing experimentally that metastatic behavior does not correlate with growth, we illustrate how progression to malignancy can occur abruptly and prior to screen detection for some tumors, which we term nascent lethal. Leveraging this model has the power to inform novel screening and interception strategies for nascent lethal cancers.

**Figure:**
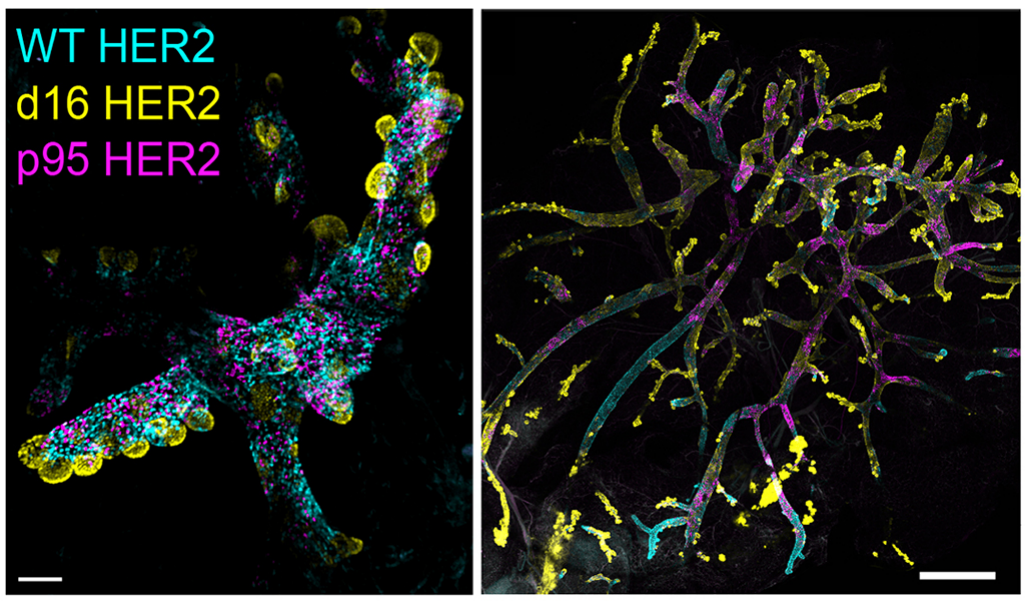
**The mammary gland before pubertal development is a small, rudimentary structure.** In our mouse model, Cre activates three fluorescently barcoded versions of HER2 before 2 weeks of age. Whole-gland imaging reveals phenotypic differences before development (2 weeks old; right) that are magnified after gland development (10 weeks old; left). Scale bar: 100 μm (left); 1 mm (right)

**Why did you choose DMM for your paper?**

This study straddled the line between cancer biology, developmental biology and mathematical modeling, while providing theoretical implications for understanding clinical cancer care. DMM offered the best audience for translational impact extracted from modeling basic biology in the mouse.

**Given your current role, what challenges do you face and what changes could improve the professional lives of other scientists in this role?**

Uncertainty is the greatest challenge I see in mine and my peers lives. The choice to pursue academic science often feels like a gamble with decreasing odds. Publication, graduation, grant funding and job searches take huge investments of time and energy with no guarantees, and, even if successful, any small mistake or misfortune presents the very real possibility of failing to achieve your goal. Throughout grad school I have seen many fellow students who are unable or unwilling to take such risk. I believe that more mechanisms that provide stability and assurance through career transitions would go a long way towards instilling confidence in the climb to success in academia.

The choice to pursue academic science often feels like a gamble with decreasing odds.

**What's next for you?**

I am currently in the process of defending my dissertation and pursuing a postdoctoral fellowship to continue chasing the mechanisms underlying cancer initiation and progression.

**Tell us something interesting about yourself that wouldn't be on your CV**

My first real interaction with a transgenic animal expressing a fluorescent protein was a GFP-expressing axolotl, which I got in college as a pet. These unique, fully aquatic salamanders were originally transduced with GFP for research in regeneration and have since made their way into the pet world. I originally picked it because I thought it would be cool to have an axolotl that glowed green, but it inspired a fascination with fluorescent proteins and transgenic animal models that has carried over into my dissertation work.
